# The surgical treatment of anomalous origin of one pulmonary artery from the ascending aorta

**DOI:** 10.1186/s13019-019-0904-0

**Published:** 2019-04-27

**Authors:** Shuo Dong, Jun Yan, Haitao Xu, Yabing Duan, Chun Liu

**Affiliations:** 1 Department of Pediatric Cardiac Surgery, National Center for Cardiovascular Disease and Fuwai Hospital, Chinese Academy of Medical Sciences, Peking Union Medical College, 167 Beilishi Road, Xicheng District, Beijing, 100037 People’s Republic of China; 2 38 Department of Cardiopulmonary Bypass, National Center for Cardiovascular Disease and Fuwai Hospital, Chinese Academy of Medical Sciences, Peking Union Medical College, 167 Beilishi Road, Xicheng District, Beijing, 100037 People’s Republic of China

**Keywords:** Anomalous origin of pulmonary artery, Congenital heart disease, Pulmonary hypertension

## Abstract

**Objective:**

This study sought to summarize the clinical experience of surgical treatment of anomalous origin of pulmonary arteries from the ascending aorta (AOPA) in Fuwai hospital.

**Methods:**

Fifty-two patients (28 males, 17.2 ± 27.2 months old and 8.7 ± 10.2 Kg weight) who have AOPA undertook surgical treatment between 1998 and 2017 were reviewed in this study, 47 out of 52 are anomalous origin of the right pulmonary artery (AORPA), among the rest of the patients are anomalous origin of left pulmonary artery (AOLPA). 27 out of 52 associate with simple cardiac abnormalities, 20 out of 52 associate with complex cardiac malformations, the remaining 5 patients without cardiac abnormalities. Among all patients who underwent surgical treatment, the direct end-to-side anastomosis strategy was applied in 26 patients, autologous pericardial-homograft patch and aortic flap were employed in 20 patients, and synthetic graft was used in 6 patients.

**Results:**

No patient died during the perioperative period. 50 out of 52 patients were followed-up for 100.1 ± 70.9 months. The rate of pulmonary arterial free restenosis for 2 years, 5 years, and 10 years is 98.0, 96.0 and 92.0%, respectively.

**Conclusions:**

The correct diagnosis and appropriate surgical treatment for AOPA could obtain excellent early and medium-term result.

## Introduction

Anomalous origin of pulmonary arteries from the ascending aorta (AOPA) is a rare congenital heart disease. It is also known as hemi-truncus, anomalous origin of the right pulmonary artery (AOPRA) is more commonly than anomalous origin of the left pulmonary artery (AOPLA). The overall morbidity of AOPA is 0.12% in congenital heart disease [1]. Pulmonary arteries originating from the ascending aorta produces a large amount of left-to-right shunt, which leads unilateral lung to receive systemic circulatory pressure. While the healthy lung receives full cardiac output from the right ventricle, resulting in pulmonary hypertension and congestive heart failure within one year after birth. As the disease is relatively rare, there are few cases reported. From 1998 to 2017, 52 cases of pulmonary artery anomalies originateing from the ascending aorta were treated in the Chinese academy of medical sciences, Fuwai hospital, and the clinical data are retrospectively analyzed.

## General information

We retrospectively analyzed the clinical data of 52 cases of pulmonary arterial abnormalities originating from ascending aorta during surgery between 1998 and 2017 in our hospital, There are 28 males and 24 females (Table 1); the mean age at the operation was 17.2 months (8 days to 19 years old). the mean weight at the operation was 8.7 kg (3 to 53 kg). All patients underwent echocardiography, 16 patients underwent angiography, and 9 patients underwent cardiac CT examination. 47 patients had an abnormal right pulmonary artery originating from the ascending aorta and 5 patients had an abnormal left pulmonary artery originating from the ascending aorta. According to Kutsche’s classification [2], 48 cases were proximal type and 4 cases were distal type. 33 cases associate with patent ductus arteriosus, 20 cases associate with atrial septal defect, 7 cases associate with ventricular septal defect, 4 cases associate with tetralogy of Fallot (4 cases of abnormal origin of left pulmonary artery), 8 cases associate with aortic-pulmonary window, and 5 cases associate with interruption of aortic arch; 5 cases did not associate with deformity. In comparison, 3 case’s diagnosis were missed before operation, no angiography or CT examination were performed before operation.

**Table 1:** Basic information of children

Item	Patient
Age(month)	17.2 ± 27.2
Weight(kg)	8.7 ± 10.2
Combined congenital heart malformation	47
Easy	27
Complex	20
Diagnosis	
AOPRA	47
AOPLA	5

*AOPRA* anomalous origin of the right pulmonary artery, *AOPLA* anomalous origin of the left pulmonary artery

## Surgical methods

Twenty-six patients were operated with the direct end-to-side anastomosis strategy. 20 patients were used autologous tissue (pericardium, aorta, pulmonary arterial) to widen or lengthen the pulmonary artery. 6 patients received anastomosis using the synthetic graft or artificial blood vessel. 1 patient who has pulmonary atresia had been missed the diagnosis before surgery, right pulmonary artery originating from the ascending aorta and left pulmonary artery absence were found during surgery, and palliative operation was made. 5 patients underwent non-extracorporeal circulation and the others underwent surgery under cardiopulmonary bypass.

Patients underwent general anesthesia and surgeries were operated under mild hypothermic cardiopulmonary bypass. Median sternotomy with extracorporeal circulation via aortic and right atrial or bicaval cannulation, and aortic occlusion with HTK antegrade cardioplegia were utilized. Through the median sternotomy, the aorta, left and right pulmonary arteries, and main pulmonary arteries are fully exposed, dissociated and released. The pulmonary arteries of abnormal origin needs to be liberated to the pulmonic hilum. If there is an patent ductus arteriosus, it needs to be ligated and cut off, which can fully free the main pulmonary artery and its branches, in order to reduce the anastomotic tension and prevent postoperative lung over-perfusion. Aortic intubation should preform as close to the distal end as possible on the ascending aorta to provide sufficient space to block the aorta. After the initiation of extracorporeal circulation, the pulmonary artery of abnormal origin is cut off and transplanted to the corresponding position of the main pulmonary artery. Anastomosis can be performed in a variety of ways: (1) If the distance is short and the tension is not large, it can be fully freed and directly anastomosed. (2) The anastomosis distance is long and the tension is large. Then use autologous tissue, such as pericardium, aorta or pulmonary arterial patch, to widen and lengthen pulmonary artery. (3) The autologous tissue cannot be used when the distance is too far away, and the pulmonary artery is lengthened with the homologous or artificial blood vessel and then anastomosed to the main pulmonary artery.

Five patients had no intracardiac deformity and were operated under non-extracorporeal circulation. 1 patient underwent operation through thoracotomy on the left side, and the other 4 underwent operations through median sternotomy. In addition to the direct end-to-side anastomosis in 1 case, the other 4 cases were far away from pulmonary artery, 3 cases used artificial blood vessels, and 1 case was connected with autologous pericardium.

## Results

In this group of patients, the extracorporeal circulation time was 96.2 ± 74 min, and the aortic blocking time was 61.1 ± 82 min.

Two patients had secondary sternotomy because of pericardial tamponade; 1 patient had right heart dysfunction on the day after operation but recovered after diuresis and peritoneal dialysis, and 1 patient who repaired the ventricular septal defect had an infective endocarditis but recovered after treated with antibiotics and was discharged from the hospital; 1 patient had a large amount of pericardial effusion in the first month after operation and was admitted to the hospital for pericardial fenestration; 4 patients had postoperative pulmonary artery stenosis, 1 patient had stent implantation and balloon dilation, 1 patient had a balloon dilation, another 2 has no special treatment and were still being followedup.

**Fig. 1:**
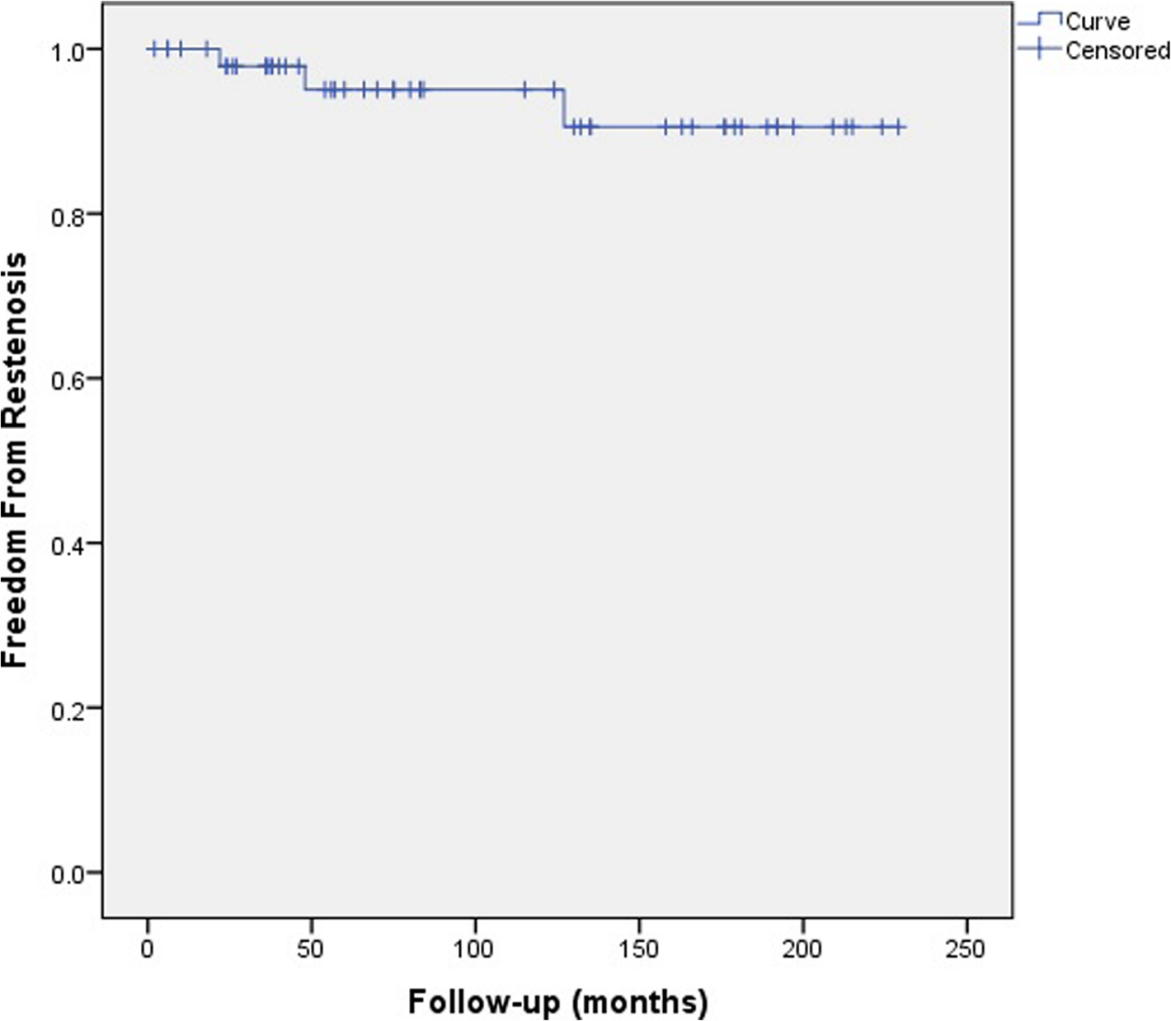
Kaplan–Meier curve demonstrating Pulmonary artery freedom from restenosis. Legend: The rate of 10 year freedom from restenosis after surgery is pretty good (>90%); direct anastomosis may be the cause of postoperative anastomotic restenosis

Fifty patients were followed up and 2 were lost. The average follow-up time was 100.1 ± 70.9 months. No death occurred during the follow-up period (Table 2). The rate of pulmonary arterial free restenosis for 2 years, 5 years, and 10 years is 98.0, 96.0, and 92.0%, respectively (Fig. 1).

**Table 2:** Child follow-up information

Item	Patient
Follow-up time(month)	100.1 ± 70.9
Death number	0
Lost number	2
Restenosis	4
Number of interventions	2

## Discussion

Anomalous origin of one pulmonary artery from the ascending aorta is a rare congenital heart disease and was first reported by Fraentzel in 1868 [3], the first successful operation was completed by Armer in 1961 [4]. Due to the low morbidity of the disease, there have been few reports. Prifti et al. reviewed nearly 30 years of literature in 2003 and a total of 127 cases of AOPA were reported [5].

There is a close relationship between AOPA and the aortic-pulmonary windows [6]. Some scholars believe that AOPA is a subtype of the latter and is called Richardson III type [7]. Clinically, APOA can be combined with aortic-pulmonary windows, coarctation of the aorta and interruption of aortic arch which is also known as Berry syndrome. In addition, CATCH22 syndrome [8] or DiGeorge syndrome [9] also have certain connections with AOPA. According to Kutsche et al., AORPA and AOLPA are different in embryonic development. AOPA can be divided into proximal and distal types [2]. Pulmonary arteries usually originate from the posterior or lateral wall of the ascending aorta, and distance 1–3 cm from the aortic valve, which are referred as the proximal type. The distal type is rarer and the ectopic pulmonary artery originates near the origin of the innominate artery. About 85% of the right pulmonary artery originated proximally, often associates with patent ductus arteriosus, ventricular septal defect, atrial septal defect, aortic-pulmonary windows, etc. While the anomalous origin of left pulmonary artery can exist alone, but more combine with Tetralogy of Fallot and right aortic arch [1].

Pulmonary arteries originate from the ascending aorta, produce a large number of left-to-right shunt, allowing unilateral lungs to receive systemic circulatory pressure while the healthy lung accepts full cardiac output from the right ventricle. With the decline of pulmonary vascular resistance during the neonatal period, more and more high-pressure blood flow directly into the pulmonary artery of abnormal origin, so that the pressure of the affected side of the pulmonary artery increases rapidly, leading to heart failure. Under the action of right ventricle volume load, vasogenic contractile factor and pulmonary vascular neurogenic reflexes, the contralateral lung artery also showed significant pulmonary hypertension. With the development of the disease, pulmonary arterial hypertension and congestive heart failure often occur within one year after birth, resulting in mortality rate up to 70% a year after birth, and 30% of patient die within three months [10]. Prifti’s statistics show that 63.6% of children undergo surgery within 6 months after birth, and most of them are in the first month [5]. Therefore, for AOPA, once diagnosed, the child should be treated as soon as possible.

According to clinical experience, echocardiography is the preferred detection method for AOPA, and the accuracy for assessing the AOPA is as high as 90% [11]. At the same time, the situation of combined cardiac malformations can also be assessed. However, there are 3 cases of missed diagnosis in this group. Therefore, When AOPA cannot be accurately diagnosed by echocardiography or there is no structural intracardiac defect but there is an unexplained pulmonary hypertension, the results of cardiac CT and angiography need to be further clarified.

With the advancement of technology, the surgical results of AOPA have achieved satisfactory results [12, 13]. According to past domestic and foreign literature, direct anastomosis is the most commonly used surgical technique [5, 9, 14]. When the abnormal pulmonary artery and the main pulmonary artery are not far from each other, and the tension of the anastomosis can be guaranteed safe after surgery, direct anastomosis is often used, especially in proximal APOA. When the anastomosis distance is too long to be directly anastomosis, a variety of methods can be used, such as artificial blood vessel connection, autogenous pericardial, to lengthen and widen the pulmonary artery. According to our experience, the direct anastomosis method will be selected only when the anastomotic distance is close enough, otherwise the autologous pericardium may be used to widen and lengthen the anterior pulmonary artery, or the aortic patch or pulmonary arterial flap methods introduced by Prifti may be used to reconstruct pulmonary artery [15]. In our early experience, we used artificial blood vessels to connect the posterior of the main pulmonary artery in children whose anastomotic distances is so long. However, due to the lack of growth potential, this blood vessel may cause long-term anastomotic stenosis. It has been rarely used in decade and there are few reports on the use of artificial blood vessels in foreign countries.

Pulmonary anastomosis stenosis is the major complication of AOPA after surgery [16]. Usually we believe that the anastomotic systolic pressure difference ≥ 50 mmHg, the average pressure difference ≥ 30 mmHg, are indications of surgical intervention. 4 cases of anastomotic stenosis occurred in this group of children. 1 of the children with AOPRA had a direct anastomosis. The right pulmonary artery systolic blood pressure difference was 58 mmHg and the flow rate was 3.8 m/s at 1 year after operation. Balloon anastomosis was performed. After that, the pressure difference was reduced to 22 mmHg; the other 1 case of AOPRA in children also used a direct anastomosis method, in the 3rd year after surgery found right pulmonary artery systolic pressure difference 38 mmHg, blood velocity 3.1 m/s; One patient had a right systolic blood pressure difference of 36 mmHg and a flow rate of 3.0 m/s 6 years after surgery. 2 children were still being followed up. In 1 case of AOPLA combined with tetralogy of Fallot, direct anastomosis was used. Anastomotic and contralateral pulmonary artery stenosis occurred gradually after surgery. Right pulmonary artery stenting and left pulmonary artery balloon dilation were performed 17 years later after surgery. No clear stenosis was observed during follow-up in other patients. The incidence of anastomotic stenosis after direct anastomosis is relatively high, and it may be a better choice to use autologous pericardium or other autologous tissue to widen and lengthen the pulmonary artery to adapt to the growth of the child’s own body. In addition, there have been reports of postoperative aortic stenosis problems [17]. According to our experience, in order to prevent stenosis caused by direct suturing when the ascending aortic incision is sutured and closed, pericardial patches and other materials may be used to expand the stumps of aorta and ectopic pulmonary artery openings.

## Conclusions

Anomalous origin of pulmonary arteries from the ascending aorta is a rare congenital heart disease. Early diagnosis and surgical treatment are effective methods to prevent irreversible pulmonary vascular disease, and detailed postoperative follow-up is a necessary way for finding postoperative anastomotic stenosis. Through effective treatment and long-term follow-up, children can obtain more satisfactory treatment effects.

## Abbreviations

AOPA: Anomalous origin of pulmonary arteries from the ascending aorta
AOPLA: anomalous origin of the left pulmonary artery
AOPRA: anomalous origin of the right pulmonary artery
CT: computed tomography

## References

[1] Cheng W, Xiao Y, Zhong Q (2008). Anomalous origin of left pulmonary artery branch from the aorta with Fallot's tetralogy[J]. Thorac Cardiovasc Surg.

[2] Kutsche LM, Van Mierop LH (1988). Anomalous origin of a pulmonary artery from the ascending aorta: associated anomalies and pathogenesis[J]. Am J Cardiol.

[3] Fraentzel SO (1868). Ein fall von abnormer communication der aorta mit der arteria pulmonalis[J]. Virchows Arch Pathol Anat.

[4] Armer RM, Shumacker HB, Klatte EC (1961). Origin of the right pulmonary artery from the ascending aorta. Report of a surgically corrected case[J]. Circulation.

[5] Prifti E, Bonacchi M, Murzi B (2004). Anomalous origin of the right pulmonary artery from the ascending aorta[J]. J Card Surg.

[6] Mori K, Ando M, Takao A (1978). Distal type of aortopulmonary window. Br Heart J.

[7] Richardson JV, Doty DB, Rossi NP (1979). The spectrum of anomalies of aortopulmonary septation[J]. Journal of Thoracic & Cardiovascular Surgery.

[8] Johnson MC, Watson MS, Strauss AW (1995). Anomalous origin of the right pulmonary artery from the aorta and CATCH 22 syndrome[J]. Ann Thorac Surg.

[9] Dodo H (1995). Anomalous origin of the left main pulmonary artery from the ascending aorta associated with Digeorge syndrome[J]. Am J Cardiol.

[10] Fontana GP, Spach MS, Effmann EL (1987). Origin of the right pulmonary artery from the ascending aorta[J]. Ann Surg.

[11] Wang J, Song Y, Cheng TO (2015). The value of transthoracic echocardiography in the diagnosis of anomalous origin of the right pulmonary artery from the ascending aorta: a single center experience from China[J]. Int J Cardiol.

[12] Goldstein BH, Bergersen L, Powell AJ (2010). Long-term outcome of surgically repaired unilateral anomalous pulmonary artery origin[J]. Pediatr Cardiol.

[13] Peng EWK, Shanmugam G, Macarthur KJD (2004). Ascending aortic origin of a branch pulmonary artery—surgical management and long-term outcome[J]. Eur J Cardiothorac Surg.

[14] Xie L, Gao L, Wu Q (2015). Anomalous origin of the right pulmonary artery from the ascending aorta: results of direct implantation surgical repair in 6 infants[J]. J Cardiothorac Surg.

[15] Prifti E, Frati G, Crucean A (2002). A modified technique for repair of the anomalous origin of the right pulmonary artery from the ascending aorta[J]. European journal of cardio-thoracic surgery: official journal of the European Association for Cardio-thoracic Surgery.

[16] Abusulaiman RM, Hashmi A, Mccrindle BW (1998). Anomalous origin of one pulmonary artery from the ascending aorta: 36 years' experience from one Centre.[J]. Cardiol Young.

[17] Kajihara N, Imoto Y, Sakamoto M (2008). Surgical results of anomalous origin of the right pulmonary artery from the ascending aorta including reoperation for infrequent complications. Ann Thorac Surg.

